# Vitamin D Controls Tumor Growth and CD8+ T Cell Infiltration in Breast Cancer

**DOI:** 10.3389/fimmu.2019.01307

**Published:** 2019-06-06

**Authors:** Esma Karkeni, Stéphanie O. Morin, Berna Bou Tayeh, Armelle Goubard, Emmanuelle Josselin, Rémy Castellano, Cyril Fauriat, Geoffrey Guittard, Daniel Olive, Jacques A. Nunès

**Affiliations:** ^1^Immunity and Cancer Team, Centre de Recherche en Cancérologie de Marseille, Equipe Labellisée Fondation pour la Recherche Médicale, Institut Paoli-Calmettes, Inserm, CNRS, Aix Marseille Université, Marseille, France; ^2^Centre de Recherche en Cancérologie de Marseille, Plateforme d'essai préclinique TrGET, Institut Paoli-Calmettes, Inserm, CNRS, Aix Marseille Université, Marseille, France

**Keywords:** immunonutrition, vitamin D, breast cancer, CD8+ T cells, inflammation

## Abstract

Women with low levels of vitamin D have a higher risk of developing breast cancer. Numerous studies associated the presence of a CD8+ T cell infiltration with a good prognosis. As vitamin D may play a key role in the modulation of the immune system, the objective of this work was to evaluate the impact of vitamin D on the breast cancer progression and mammary tumor microenvironment. We show that vitamin D decreases breast cancer tumor growth. Immunomonitoring of the different immune subsets in dissociated tumors revealed an increase in tumor infiltrating CD8+ T cells in the vitamin D-treated group. Interestingly, these CD8+ T cells exhibited a more active T cell (T_EM/CM_) phenotype. However, in high-fat diet conditions, we observed an opposite effect of vitamin D on breast cancer tumor growth, associated with a reduction of CD8+ T cell infiltration. Our data show that vitamin D is able to modulate breast cancer tumor growth and inflammation in the tumor microenvironment *in vivo*. Unexpectedly, this effect is reversed in high-fat diet conditions, revealing the importance of diet on tumor growth. We believe that supplementation with vitamin D can in certain conditions represent a new adjuvant in the treatment of breast cancers.

## Introduction

Breast cancer is the most frequently diagnosed cancer and the leading cause of cancer-related death in women worldwide (1). It is now well established that the immune system plays an important role in disease outcome in patients affected with cancer. However, the assessment of immune response to the tumor in individual patients is difficult (2). Malignant tumors are associated with changes of peripheral blood lymphocyte phenotype and function (3, 4). However, it is obvious that changes detected in the peripheral blood may not necessarily reflect the situation in the tumor microenvironment. A large body of evidence suggests that the main events determining the outcome of the host-tumor relationship occur at the tumor site (2). A number of parameters have been assessed as biomarkers of the host immune response in the tumor microenvironment (5–7). The presence of tumor-infiltrating lymphocytes (TILs) has been recognized as a biomarker of anti-tumor immune response across a wide range of tumors. The presence of TILs has been associated with improved prognosis in epithelial ovarian carcinoma (8), endometrial cancer (9, 10), and also breast cancer (11–13). It was demonstrated that TILs obtained from patients with breast cancer, exhibited cytolytic activity against tumor cells (14, 15). An Immunoscore based on the combined analysis of CD8+ plus CD45RO+ T cells in specific tumor regions is a useful criteria for the prediction of tumor recurrence and survival in patients with early stage colorectal cancer (16). Moreover, a correlation between tumor infiltrating CD8+ T lymphocytes in invasive breast cancer and better prognosis has been demonstrated.

Behaviors such as reduction in alcohol intake and consumption of fats and red meat, together with an increase in the amount of fibers and vitamin D in the diet, may be direct or indirect protective factors against breast cancer. Vitamin D (VD; cholecalciferol) is mostly known for its major role in bone metabolism and calcium homeostasis (17), but accumulating evidence also suggests that VD is recognized as an immune modulator and has positive effects in the prevention and treatment of cancer (18). Several epidemiologic studies suggest inverse correlations between serum 25-hydroxyvitamin D (25(OH)D) levels and breast cancer development, risk for breast cancer recurrence, and mortality in women with early-stage breast cancer (19–21). Mouse model studies have shown that VD or its biologically active metabolite, calcitriol (1,25(OH)2D3) can inhibit cellular proliferation and angiogenesis and induce differentiation and apoptosis (22, 23). VD modulates its biological effects by directly regulating target gene expression through the vitamin D receptor (VDR), a ligand-regulated transcription factor and a member of the nuclear receptor superfamily. A recent study has shown that VD, through its nuclear binding to VDR, regulates cell death by modulating autophagy in luminal-like breast cancer-cell models, and also in normal mammary gland in mice (24). The indirect biological effects induced by VD in the tumor microenvironment are poorly investigated.

Some studies have indicated that diet may have an influence in approximately 35% of breast cancer cases (25). Weight gain since early adulthood and obesity have been associated with increased post-menopausal breast cancer risk in several prospective studies (26–29). Indeed, obesity favors an increase in circulating estrogens and entails increased risk of hormone-dependent breast cancer (30–32). The pathway of chronic inflammation (pro-inflammatory cytokines) induced by adipose tissue also contributes to both tumor transformation and resistance to therapy (33). It is known that the balance between pro-inflammatory and anti-inflammatory T cells is modified during obesity-induced inflammation in visceral adipose tissue, the pool of pro-inflammatory T cells such as CD4+ and CD8+ T cells is increased, and anti-inflammatory T cells such as Tregs are decreased in diet-induced obesity (34). Moreover, macrophages in obese tissue are mostly M1 macrophages (also known as classically activated macrophages), which secrete pro-inflammatory cytokines such as interleukin (IL)-6 and tumor necrosis factor (TNF)-α (35–37).

Our work aimed to explore the effects of VD on the breast cancer progression in mice from basal to overweight conditions and its consequences on CD8+ T cell infiltration into the tumor. Our data provide evidence that, depending of diet conditions, the VD supplementation has opposite effects on both in CD8+ T cell infiltration and tumor growth.

## Materials and Methods

### Animal Experiments

In accordance with European Directive 2010/63/EU on the use of animals for scientific purposes, the experimental protocol was approved by an Institutional Animal Care and Use Committee. The corresponding Project Authorization (agreement No. 11868-2017101916238361) was delivered by the French Ministry of Research and Higher Education. 6-week-old female C57BL/6J mice were obtained from Janvier Labs (France). The animals were maintained at 21°C under a 12 h light, 12 h dark cycle with a 55% humidity level.

### EO771 Tumor Cell Implantation

EO771 cell line, a spontaneously developing breast adenocarcinoma from C57BL/6 mice, was purchased from CH3 BioSystems LLC (Amherst, NY, USA). EO771-GFP+-Luciferase were generated by transduction of GFP-Luciferase lentiviral vector (pRRL-GFP/Luc2 vector), cells were maintained in *RPMI*-1640 media supplemented with 10% FCS.

To establish orthotopic implantation of breast tumors, EO771-Luc/GFP cells were suspended in 100 μL of a mixture of PBS/Matrigel (v/v) (Corning). 2 × 10^5^ EO771-Luc/GFP cells were injected into the 4th inguinal mammary of the two fat pads of 7-week-old female C57BL/6 mice. Tumor growth was monitored by caliper measurements. To avoid tumor necrosis, and in compliance with regulations for use of vertebrate animals in research, animals were euthanized before the tumors reached 1,500 mm^3^.

### VD Supplementation in Standard Diet Condition

To assess the impact of VD on breast cancer progression in basal conditions, the mice (*n* = 8–10) were fed *ad libitum* with standard diet (AIN-93M, 10% energy from lipids) (Safe, Augy, France) and received by gavage the native form of VD (cholecalciferol) (40 International Units (IU)/day per mouse; Sigma Aldrich) (Vitamin D group) or vehicle alone (olive oil) (control group) seven times in 2 weeks. As a fat-soluble vitamin, VD was diluted in olive oil to ensure better absorption, as previously described (38–40). VD supplementation was performed seven days after cell injection.

### VD Supplementation in High-Fat Diet Conditions

To assess the impact of VD on breast cancer progression on inflammatory conditions associated with overweight, the mice (*n* = 8) were fed *ad libitum* with high-fat diet (245-HF, 45% energy from lipids) (Safe, Augy, France). After 8 weeks of high-fat diet, mice were injected with EO771 cells. Seven days after cell injection, mice received VD (cholecalciferol) (40 IU)/day per mouse; Sigma Aldrich) (Vitamin D group) or vehicle alone (olive oil) (control group) seven times in 2 weeks. This VD concentration (40 IU of VD per mouse) has been reported as being non-toxic to rodents, without major risk of hypercalcemia (38, 41).

### CD8+ T Cell Depletion

To study the impact of the depletion of CD8+ T cells on EO771 tumor growth, CD8+ T cells were depleted by 11 successive injections of the 53–5.8 mAb (BioXCell) reacting with mouse CD8β in female C57BL/6 mice. Then, 2 × 10^5^ EO771-Luc/GFP cells were injected in the fat pad of the mammary glands (*n* = 12). Tumor growth was monitored by caliper measurements.

### Tumor Dissociation

Mammary tumors were dissociated mechanically and enzymatically using collagenase/hyaluronidase (StemCell Technologies, Grenoble, France) digestion in a water bath under slight agitation at 37°C for 30 min, to generate single-cell suspensions for flow cytometry staining. To study Granzyme B production (intracellular staining after fixation and permeabilization), samples were placed in the presence of Golgi transport inhibitors (Golgi stop/plug) (BD Biosciences, Le Pont-De-Claix, France) during the entire process of cell extraction.

### Isolation of Stromal Vascular Fraction

Stromal-vascular cells were isolated from visceral adipose tissue using *Clostridium histolyticum* collagenase (Sigma Aldrich, Saint-Quentin Fallavier, France) as previously described (38). The samples were incubated at 37°C under shaking conditions until complete digestion. After filtration on a 70 μm sieve (Corning), samples were centrifuged for 10 min at 400 g. The pellet (stromal vascular fraction) was used for flow cytometry analysis and the upper phase (adipocytes) was used to quantify inflammatory markers.

### RNA Isolation and Real-Time PCR

Total cellular RNA was extracted using TRIzol reagent (Life Technologies, Villebon-sur-Yvette, France) according to the manufacturer's instructions. cDNAs were synthesized from 1 μg of total RNA using random primers and Moloney murine leukemia virus reverse transcriptase (Life Technologies, Villebon-sur-Yvette, France). Real-time quantitative RT-PCR analyses were performed using the Power SYBR Green PCR Master Mix (Life Technologies, Villebon-sur-Yvette, France) and detected with a CFX96 Real-Time PCR Detection System (Bio-Rad). For each condition, the expression was quantified in duplicates and the ribosomal protein 18S mRNA was used as the endogenous control in the comparative cycle threshold (C_T_) method (2 ^−(ΔΔCT)^). The respective sets of primers sequences are 18S-F_cgccgctagaggtgaaattct and 18S-R_cattcttggcaaatgctttcg (for 18S mRNA); Il6-F_acaagtcggaggcttaattacacat and Il6-R_ttgccattgcacaactcttttc (for *Il6* gene); Ccl2-F_catccacgtgttggctca and Ccl2-R_gatcatcttgctggtgaatgagt (for *Ccl2* gene) Ccl5-F_tgcagaggactctgagacagc and Ccl5-R_gagtggtgtccgagccata (for *Ccl5* gene); Ccl11-F_agagctccacagcgcttct and Ccl11-R_gcaggaagttgggatgga (for *Ccl11* gene); Cx3cl1-F_catccgctatcagctaaacca and Cx3cl1-R_cagaagcgtctgtgctgtgt (for *Cx3cl1* gene), Adiponectin- F_tcctggagagaagggagagaaag and Adiponectin-R_tcagctcctgtcattccaaca (for *Adiponectin* gene); Pparg-F_caagaataccaaagtgcgatcaa and Pparg-R_gagctgggtcttttcagaataataag (for *Pparg* gene); Cebpa-F_agcaacgagtaccgggtacg and Cebpa-R_tgtttggctttatctcggctc (for *Cebpa* gene); Pgc1a-F_ ttccaaaaagaagtcccatacaca; and PGC1a-R_gataaagttgttggtttggcttga (for *Pgc1a* gene).

### Flow Cytometry Analysis

Tumor, stromal vascular fraction, spleen and tumor-draining lymph nodes were used for flow cytometry analysis. Before labeling, cells were incubated in Fc-blocking (CD16/32 antibody). Cells were labeled with fluorescent antibodies against CD3, CD4, CD8, CD44, CD62L, PD-1, CD11b, CD11c, F4/80, Granzyme B (intracellular staining after fixation and permeabilization). Antibodies were purchased from (Life Technologies, Villebon-sur-Yvette, France), Biolegend (San Diego, CA, USA) and BD Biosciences (Le Pont-De-Claix, France).

All data were acquired on a FACS FORTESSA 3 lasers flow cytometer (Becton–Dickinson, Le Pont-De-Claix, France) and analyzed with FlowJo software (Tree Star, Ashland, OR, USA).

### Analysis of Plasma Samples

Plasma 25(OH)D concentrations were measured using an ELISA kit (Promokine, Promocell). IL-6 cytokine was quantified with Ready-SET-Go! ELISA (Life Technologies, Villebon-sur-Yvette, France). CCL5 chemokine was quantified with Instant ELISA kit (Life Technologies, Villebon-sur-Yvette, France).

### Statistical Analysis

The data were expressed as the mean ± SEM. Prism 5.03 software (GraphPad, San Diego, CA, USA) was used for all statistical analysis. Statistical significance between control and VD groups was determined by two-tailed Student's *t*-test. ^*^P < 0.05, ^**^P < 0.01, ^***^P < 0.001.

## Results

### Vitamin D Limits Breast Cancer Tumor Progression

**Figure 1:**
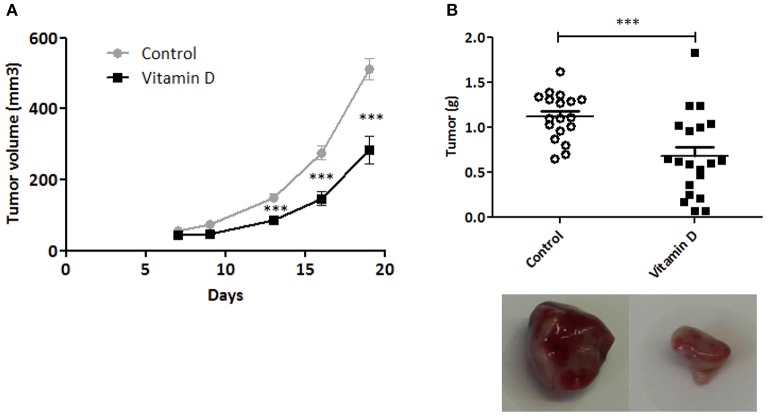
Vitamin D limits EO771 tumor progression in mice. EO771 cells were injected in the fat pad of the mammary glands (*n* = 8–10) of female mice fed with standard diet. **(A)** Tumor size was measured with a caliper. The values of tumor volume (mm^3^) are reported (Mean ± SEM). **(B)** Tumors were weighed at the end of the experiment (g). Statistical significance between control and VD groups was determined by two-tailed Student's *t*-test. ^***^P < 0.001.

To control the efficiency of VD supplementation, 25(OH)D was measured in the serum of mice at the end of the experiment (Supplemental Figure 1). VD decreased tumor growth starting at day 13 post injection and this difference was conserved until mouse sacrifice (day 20) [VD gavage (284 ± 38.9 mm^3^) compared with control mice (512 ± 31.2 mm^3^, *P* < 0.001)] (Figure 1A). In parallel, we detected a decrease in tumor weight (1.6-fold compared with control group) (Figure 1B). Altogether, these data show that VD supplementation is sufficient to reduce tumor growth.

### Vitamin D Promotes CD8+ T Cell Tumor Infiltration

Numerous studies and more recent pre-clinical or clinical data have highlighted the important role of cytotoxic T cells in breast cancer, especially with respect to immune-checkpoint immunotherapies. Therefore, we wondered whether VD had an impact on CD8+ T cells in our model.

**Figure 2:**
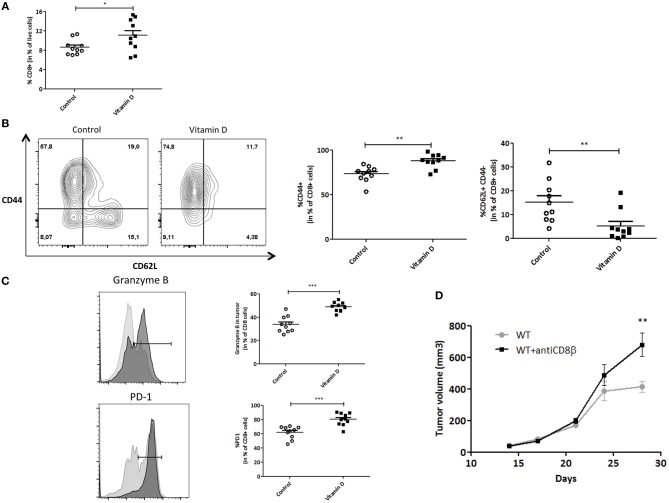
Vitamin D modulates the CD8+ T cell phenotype in the tumor in basal conditions. Flow cytometry analysis of the tumor from Control and Vitamin D-treated mice. **(A)** Quantification of the CD8+ T cells. **(B)** Quantification of the CD8+ T cell markers (CD44, CD62L) **(C)** and Granzyme B and PD-1. **(D)** CD8+ T cells were depleted by the injection of CD8β mAb in C57BL/6J (WT) mice. Then, EO771 cells were injected in the fat pad of the mammary glands (*n* = 12). Tumor size is measured with a caliper. The values of tumor volume (mm^3^) are reported (Mean ± SEM). Statistical significance between control and VD groups was determined by two-tailed Student's *t*-test. ^*^P < 0.05, ^**^P < 0.01 and ^***^P < 0.001.

After tumor dissociation, we performed immunomonitoring to study the different immune populations. We observed a significant increase of tumor infiltrating CD8+ T cell frequency (Figure 2A). We next studied T cells subsets. We clearly saw a decrease in the naïve T cells (CD62L+) population in TIL CD8+ cells when VD was present. This observation is correlated with an increase of CD44+ T cell subsets (central and effector memory cells; Figure 2B). To further investigate the activation status of the CD8+ T cells that infiltrated the tumor, we assessed the cytotoxic T cell phenotype by using a specific marker, Granzyme B, and for activated T cell phenotype, PD-1. Indeed, VD-supplemented mice exhibited higher Granzyme B and PD-1 expression levels in tumor infiltrating CD8+ T cells (1.3 and 1.4-fold, respectively) as compared with control mice (Figure 2C). Our results argue for an increased T cell recruitment to the tumor site and T cell activation in VD context compared to control.

Finally, to further characterize the EO771 model and its sensitivity to CD8+ T cells, these cells were depleted by the injection of CD8β mAb. CD8+ T cell depletion exacerbates tumor growth (680 ± 75 mm^3^) compared with control mice (413 ± 34 mm^3^, *P* < 0.01) (Figure 2D), emphasizing the importance of CD8+ T cells in controlling tumor growth.

### Vitamin D Modulates Pro-Inflammatory Macrophage Infiltration in Peripheral Tissues, but Not Inside the Tumor

**Figure 3:**
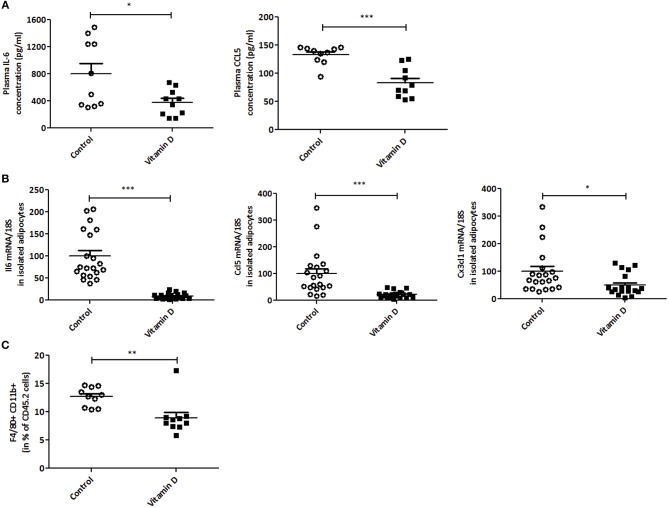
Vitamin D limits inflammation in plasma, adipocytes and stromal vascular fraction of mice. **(A)** In EO771 breast cancer model (Standard diet), inflammatory cytokines (IL-6 and CCL5) levels were measured in plasma by ELISA (*n* = 8–10). **(B)** mRNA levels of Il6, Ccl5 and Cx3cl1 were quantified through qPCR in isolated adipocytes from visceral adipose tissue (*n* = 10) and expressed relative to 18S ribosomal RNA. The data are expressed as relative expression ratios. **(C)** Inflammatory macrophages (F4/80+CD11b+) were quantified in stromal vascular fraction of adipose tissue (*n* = 8–10) by flow cytometry. The values are presented as the mean ± SEM. ^*^P < 0.05, ^**^P < 0.01, ^***^P < 0.001 compared with control group.

**Figure 4:**
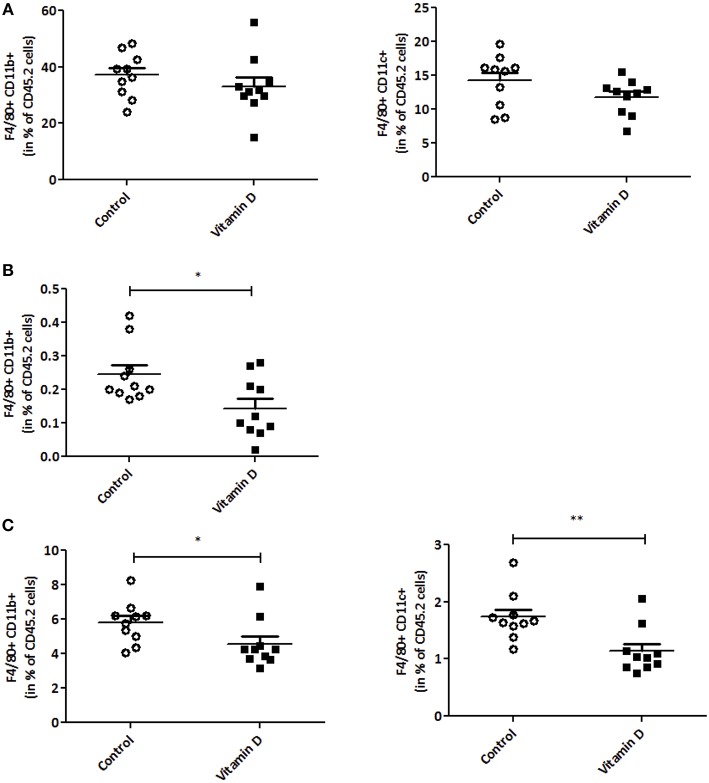
Vitamin D modulates macrophage infiltration. Frequencies of macrophages “F4/80+CD11b+” and “F4/80+CD11c+” were analyzed by flow cytometry in tumor **(A)**, tumor-draining lymph nodes **(B)** and spleen **(C)** (*n* = 10). The values are presented as the mean ± SEM. ^*^P < 0.05, ^**^P < 0.01 compared with control group fed with standard diet.

Since VD is known to regulate inflammation (38, 42), we next decided to investigate this in our tumor model. A decrease in IL-6 and CCL5 protein secretion was observed after VD supplementation in mice (2.1 and 1.6-fold, respectively) (Figure 3A). To further characterize this, we studied the effect of VD on cytokine/chemokine gene expression in isolated adipocytes from the visceral adipose tissue. As shown in Figure 3B, the levels of *Il6, Ccl5*, and *Cx3cl1* transcripts were significantly decreased (11, 4.5, and 2-fold, respectively) after VD supplementation. In addition, flow cytometry was used to detect pro-inflammatory macrophages in the stromal vascular fraction of adipose tissue. We observed a reduction in M1 macrophages (F4/80+CD11b+) population in mice supplemented with VD (1.42-fold compared with control mice) (Figure 3C) whereas no modification was observed in macrophage infiltration into the tumor (Figure 4A). We also studied these markers in lymphoid organs like tumor-draining lymph nodes and spleen. In lymph nodes, we observed a reduction in F4/80+CD11b+ macrophage infiltration (1.4-fold compared with control mice) (Figure 4B). Finally, VD reduced F4/80+CD11b+ and F4/80+CD11c+ macrophage levels in spleen of mice supplemented with VD (1.3 and 1.5-fold, respectively) (Figure 4C). We next evaluated the effect of VD supplementation on adipogenesis. For this purpose, the mRNA levels of adiponectin, peroxisome proliferator-activated receptor gamma (Pparg), PPARγ-coactivator 1α (Pgc1a) and CCAAT enhancer binding protein-α (Cebpa) were quantified by RT-qPCR. We have shown that VD supplementation in standard diet condition significantly limits adiponectin expression in adipocytes with a tendency for a decrease in Pparg, Pgc1a and Cebpa (Supplemental Figure 2).

Our data show a reduction of pro-inflammatory cytokines and macrophages (M1) in accordance with previously published data.

### Vitamin D Increases EO771 Tumor Progression in Mice Subjected to a High-Fat Diet

Since obesity increases tumor progression, we next wondered whether VD would reverse overweight-related inflammation and reduce tumor progression, similarly to basal diet conditions. Mice were first fed for 8 weeks with a high-fat diet (45% energy from lipids) leading to a significant gain of weight (Supplemental Figure 3A) and a reduction of 25(OH)D compared with control mice (Supplemental Figure 3B). EO771 were then injected as in previous experiments, and VD was administrated 1 week after tumor cell transplant.

**Figure 5:**
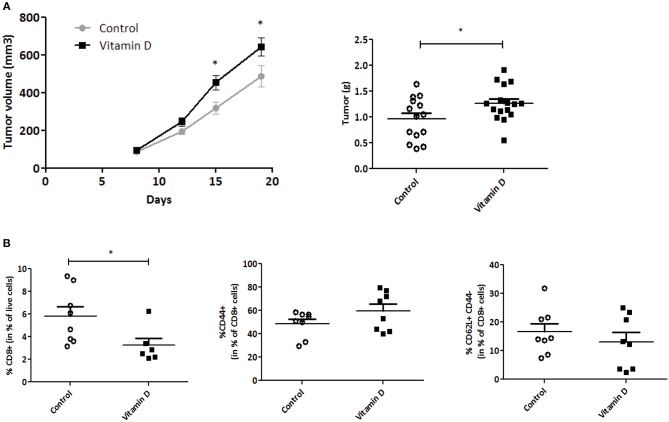
Vitamin D increases EO771 tumor progression in overweight mice. EO771 cells were injected in the fat pad of the mammary glands (*n* = 8) of female mice fed with high-fat diet. **(A)** The tumor size was measured with a caliper. The values of tumor volume (mm^3^) are reported (Mean ± SEM). **(B)** Tumors were weighed at the end of the experiment (g). **(C)** CD8+ T cells and their markers were quantified by flow cytometry. Statistical significance between control and VD groups was determined by two-tailed Student's *t*-test. ^*^P < 0.05.

Similarly to standard fat diet, VD administration resulted in increase of plasmatic 25(OH)D concentration (Supplemental Figure 4A). VD administration also resulted in a lower systemic inflammation with reduced IL-6 and CCL5 plasmatic concentrations (Supplemental Figure 4A), reduced Il6, Ccl2, Ccl5, Cx3cl1, and Ccl11 proinflammatory cytokine mRNA expression in visceral adipocytes (Supplemental Figure 4B) and no modification of adipogenic markers (Supplemental Figure 4C). Surprisingly, in contrast to basal diet conditions, VD supplementation of overweight-mice resulted in a faster tumor progression. Hence VD significantly increased tumor growth starting at day 15 post injection. Prior to sacrifice, a higher tumor volume was observed in obese mice that received VD gavage (642 ± 45 mm^3^) compared with control mice (486 ± 53 mm^3^, *P* < 0.05) (Figure 5A). The increase of tumor volume correlated with the increase in tumor weight (1.4-fold compared with control group) (Figure 5B). We next evaluated the infiltration of CD8 T cells in the tumor. Unexpectedly, we observed in VD-supplemented overweight mice a reduction of infiltrating CD8 T cells compared with control mice (Figure 5C) but no modification in the CD62L/CD44 T cell subsets. When studying macrophage population, we observed an increase of F4/80+CD11b+ and F4/80+CD11c+ macrophages in tumor of obese mice supplemented with VD (Supplemental Figure 5A). We also assessed macrophage frequency in other tissues. We showed that there is an increase of M1 macrophages in the tumor-draining lymph nodes (Supplemental Figure 5B). Finally, VD reduced F4/80+CD11c+ macrophage levels in spleen as observed in basal conditions (Supplemental Figure 5C).

## Discussion

In the present study, using female mice injected with EO771 breast cancer cells and supplemented with VD, we report a beneficial role of VD on tumor growth progression and inflammation. Indeed, VD supplementation reduced tumor weight and increased CD8+ T cell infiltration into the tumor. Interestingly, these CD8+ T cells exhibited a more active central/effector memory T cell phenotype, which are considered to have increased anti-tumor properties (43). These data are in agreement with data that highlight TILs as an important predictive and prognostic biomarker in patients with breast cancer (44). Ono et al. observed an association between pathological response and TIL score in patients with triple-negative breast cancer, but not in patients with other tumor types (45). Therefore, a general consensus emerged on the central role played by effector T cells in the anti-tumor response. Similar results were obtained in colorectal cancer in which quantification of immune cell densities revealed the major positive role of cytotoxic and memory T cells for patient survival (46). The present study indeed also reports that CD8+ T cells infiltrating the tumor harbor an activated/cytotoxic phenotype eliciting PD-1/Granzyme B expression. In line with these results, several studies on cancer patients have reported that PD-1 expression is highly expressed on tumor infiltrating CD8+ T cells in various immunogenic tumors (47–49).

**Figure 6:**
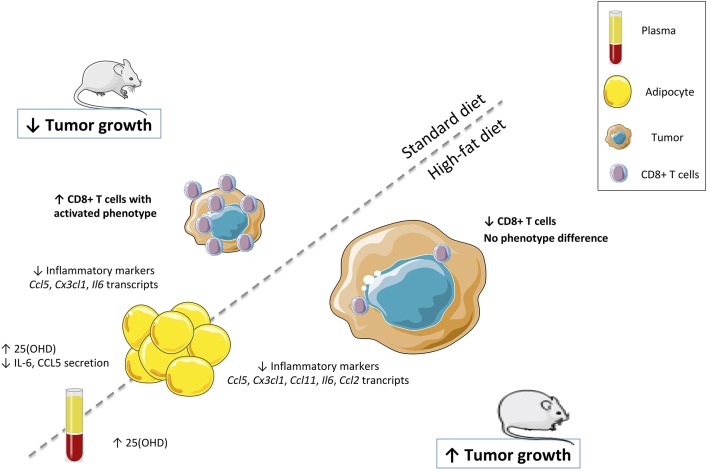
Vitamin D controls breast cancer tumor growth and CD8+ T cells infiltrating the tumor. VD supplementation was validated in both models by the quantification of the 25(OH)D levels and inflammatory cytokines (IL-6 and CCL5) in plasma and mRNA levels of inflammatory markers (*Il6, Ccl5, Ccl2, Ccl11*, and *Cx3cl1*) in adipocytes. Tumor size was measured and CD8+ T cells were quantified in the tumor.

Surprisingly, in contrast to standard diet condition, VD supplementation of overweight-mice resulted in increased tumor progression without CD8+ T cell infiltration (illustrated in Figure 6). The impact of VD supplementation on inflammation associated with obesity has been largely described. It has been reported a beneficial role of VD supplementation on weight gain limitation in mice (50, 51). Indeed, such supplementation reduced the weight gain induced by high-fat diet and improved insulin sensitivity. The reduced weight gain in VD supplemented mice is primarily due to a limitation of fat mass accumulation (50). We have also shown in a previous study that VD displays immunoregulatory effects and reduces adipocyte inflammation *in vitro* and *in vivo*. Indeed, VD supplementation decreases the expression and secretion of a large range of inflammatory markers and macrophage infiltration in the adipose tissue of high-fat diet fed mice (38). In the present study, we report a beneficial role of VD supplementation on inflammation both in basal or overweight conditions. We have shown that VD is able to limit the expression of inflammatory markers in adipocytes (Il6, Ccl5, and Cx3cl1) and their secretion (IL-6 and CCL5) in the plasma. These results are in agreement with the previously reported anti-inflammatory effects of VD on adipocytes (38, 52). Taken together, our results reveal the influence of diet on tumor growth control and CD8+ T cell infiltration by VD supplementation. The difference observed between normal and high-fat diet conditions could be explained by a modification in the VD metabolism in adipocytes, in particular Cyp27a1 in overweight mice, which is involved in the first hydroxylation of cholecalciferol (data not shown). This enzyme is known to be increased in adipose tissue of obese mice, which suggests the possible increase of 25-hydroxylation in adipose tissue, resulting in higher local production of 25(OH)D in adipose tissue (53). Several studies about obesity have demonstrated that 25(OH)D is diluted in a higher volume in obese patients (54). We hypothesized that the increase of adipocytes in overweight-mice reduces 25(OH)D in plasma, which could have an impact on a decreased CD8+ T cell infiltration.

We next wondered if the observed contrasting effects could be due to a modification of adipogenic markers under VD supplementation. Using RT-qPCR approach, we found that VD limited adiponectin expression and a tendency for a decrease in Pparg, Pgc1a, and Cebpa without modification in high-fat condition. Our results obtained in normal diet fed-mice are in accordance with previous results reported in mouse models (55) but they cannot explain the difference between the two conditions. Indeed, the effects of VD on adipogenesis have been analyzed in several animal models, and the majority of these studies suggest that VD plays an inhibitory role in adipogenesis. The few cell culture and supplementation studies that have investigated adipogenesis in human cells indicate that, in contrast to findings from rodent studies, VD is proadipogenic (56, 57). The difference in results among mouse and human studies could be due to differences in diet, treatment periods, cell types, and/or VD treatment protocols.

Differences on CD8+ T cell infiltration into the tumor of standard diet and high-fat diet-fed mice could finally be explained by the decrease of expression levels of chemokine receptor CCR7 which is expressed on central memory T cells and involved in chemotaxis (58). Further studies are needed to investigate the molecular mechanisms involved in the regulation of CD8+ T cell migration inside the tumor under different kinds of diets.

A CD8+ T cell infiltrate is generally associated with a good cancer patient prognosis. However, other immune cell infiltrates are associated with a poor patient prognosis [infiltrates with immunosuppressive cell types such as regulatory T cells (Tregs), tumor-associated macrophages (TAMs) or myeloid-derived suppressor cells (MDSCs)] (59). In this study, only CD4+CD25+CD127low/- T cell subset has been considered (Supplemental Figure 6), as this subset could contain some Tregs. Flow cytometry analysis showed that VD did not affect the presence of CD4+CD25+CD127low/- T cells in the tumor both in basal and high-fat conditions. It would be of interest to further analyze, in the tumor microenvironment, these cell types harboring immunosuppressive properties: among immune cells but also in other cell lineages such as the cancer-associated fibroblasts (CAFs).

Adipose tissue is increasingly recognized as an active endocrine organ, which secretes several adipose tissue-specific hormones including adipokines. It plays a significant role in the regulation of homeostasis. Importantly, tumor progression does not necessarily depend on fat mass, but may be influenced more significantly by the specific metabolic state of the local adipocyte population (60). Adipose tissue is altered during inflammatory context related to obesity (61) and breast cancer (62). The factors inducing leukocyte and chemokine infiltration in adipose tissue are probably numerous. The paracrine, autocrine and endocrine signals appear to be responsible for this phenomenon (63). Indeed, adipose tissue and adipocytes in particular produce and release a variety of adipokines notably chemokines which are defined as “cytokines with selective chemoattractant properties,” coordinating leukocyte movement to sites of inflammation or injury (64). Most of the breast body is made up of adipose tissue which contains both adipocytes and immune cells. In following studies, it will be important to combine immunomonitoring to adipocyte behavior studies.

The clinical data related to the effect of VD supplementation on breast cancer are still unclear. Indeed, only a few VD supplementation trials with breast cancer have been conducted, and these have suffered from major limitations (as inappropriate dose…) (65). Another recent multicentric, randomized, placebo-controlled trial, with a two-by-two factorial design, of vitamin D3 (cholecalciferol) and omega-3 fatty acids for the prevention of cancer, was conducted in the United States. Supplementation with VD did not result in a lower incidence of invasive cancer than placebo (66). Given the interest in using VD to reduce cancer risk in lean patients, further research is needed, particularly randomized controlled trials, to demonstrate in humans whether individuals with low levels of circulating 25(OH)D are at increased risk of developing cancer (VD in prevention strategy) and whether VD supplements can reduce cancer risk and progression and improve outcome (VD in therapy). In conditions where VD favors access for cytolytic T cells to the tumor site, VD supplementation (T-cell migration inducer) could be combined with an immune checkpoint immunotherapy (T-cell activation inducer) in the treatment of breast cancers.

In conclusion, our results show that VD modulates tumor growth progression and the infiltration of the cytotoxic CD8+ T cells in the tumor of mice depending on the type of diet. If our data can be confirmed in human in randomized clinical trials, VD supplementation could represent a new adjuvant in the treatment of breast cancers.

## Ethics Statement

In accordance with European Directive 2010/63/EU on the use of animals for scientific purposes, the experimental protocol was approved by an Institutional Animal Care and Use Committee. The corresponding Project Authorization (agreement No. 2017101916238361) was delivered by the French Ministry of Research and Higher Education.

## Author Contributions

EK, GG, SOM, RC, and JAN designed the study, performed the experiments and analyzed the results. AG, EJ, BBT, CF, and DO assisted with realization and interpretation of the experiments. JAN supervised and directed the research. EK and JAN wrote the manuscript. All authors discussed the results and commented on the manuscript.

### Conflict of Interest Statement

DO is the cofounder and shareholder of Imcheck Therapeutics. The remaining authors declare that the research was conducted in the absence of any commercial or financial relationships that could be construed as a potential conflict of interest.
