# Supplementary material for: Vitamin D Controls Tumor Growth and CD8+ T Cell Infiltration in Breast Cancer
Source: Front Immunol. 2019 Jun 6;10:1307. doi: 10.3389/fimmu.2019.01307 (PMC6563618; doi:10.3389/fimmu.2019.01307)
Supplement: Supplementary file 1 [file Data_Sheet_1.docx]

**Supplemental Figure 1**

**Vitamin D** **supplementation increases 25(OH)D levels in EO771 mice.** 25(OH)D levels were measured in plasma by ELISA (n = 10). The data are expressed as relative expression ratios. The values are presented as the mean ± SEM. *** *P* < 0.001 compared with control group.

**Supplemental Figure 2**

**
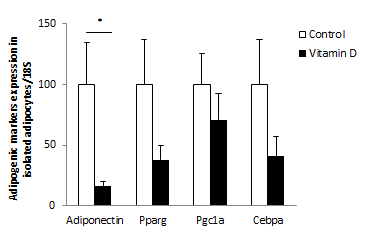
**

**Effect of VD supplementation on expression of adipogenic markers in adipocytes in EO771 mice.** The mRNA levels of *adiponectin, Pparg, Pgc1a* and *Cebpa* were quantified through qPCR in isolated adipocytes in mice subjected to standard diet. The data are expressed as relative expression ratios. Control condition was set as 100%. The values are presented as the mean ± SEM. * *P <* 0.05 compared with control group.

**Supplemental Figure 3**

A

B

**
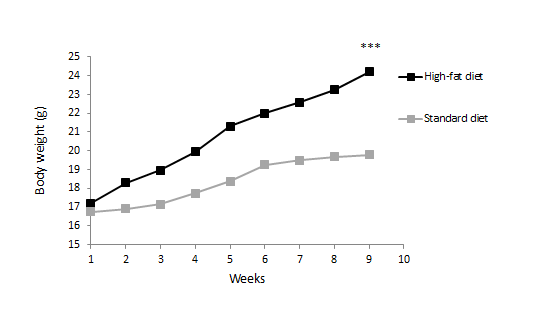
**

**High-fat diet induces significant weight gain and a reduction of 25(OH)D levels in mice within 8 weeks.** The mice were fed *ad libitum* with high-fat diet (245-HF, 45% energy from lipids) for 8 weeks. Body weight evolution was quantified for each mouse (n = 8) **(A)**. 25(OH)D levels were measured in plasma by ELISA (n = 8) **(B)**. The values are presented as the mean ± SEM. *** *P* < 0.001 compared with group fed *ad libitum* with standard diet (AIN-93M, 10% energy from lipids).

**Supplemental Figure 4**

A

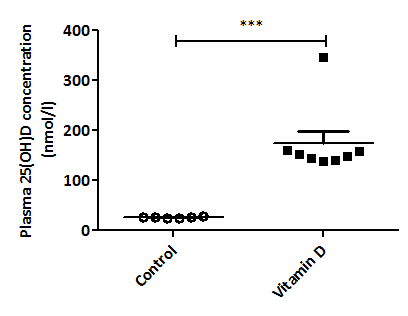


C

B


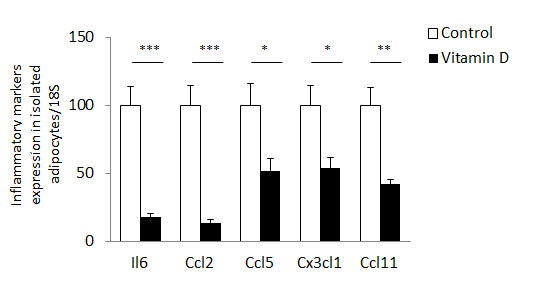


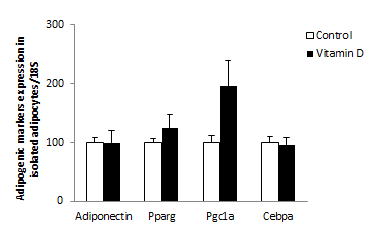


**Vitamin D** **limits inflammation in plasma and adipocytes in EO771 mice subjected to high-fat diet conditions.** In EO771 breast cancer model (high-fat conditions), the 25(OH)D levels and inflammatory cytokines (IL-6 and CCL5) were measured in plasma by ELISA (n = 8) **(A)**. The mRNA levels of inflammatory markers (*Il6*, *Ccl2*, *Ccl5*, *Ccl11* and *Cx3cl1*) **(B)** and adipogenic markers (*adiponectin, Pparg, Pgc1a* and *Cebpa*) **(C)** were quantified through qPCR in isolated adipocytes (n = 8 per group) and expressed relative to 18S ribosomal RNA. The data are expressed as relative expression ratios. Control condition was set as 100%. The values are presented as the mean ± SEM. * *P <* 0.05, ** *P <* 0.01, *** *P* < 0.001 compared with control group.

**Supplemental Figure 5**

A

Tumor

B

Lymph nodes

C

Spleen

**Vitamin D** **modulates macrophage infiltration in EO771 mice subjected to high-fat diet conditions.** Macrophages ‘’F4/80+CD11b+’’ and ‘’F4/80+CD11c+’’ were analysed by flow cytometry in tumor **(A)**, tumor-draining lymph nodes **(B)** and spleen **(C)** (n = 6-8). The values are presented as the mean ± SEM. * *P <* 0.05, ** *P <* 0.01 compared with control group fed with high-fat diet.

**Supplemental Figure 6**

**CD8**

**CD4**


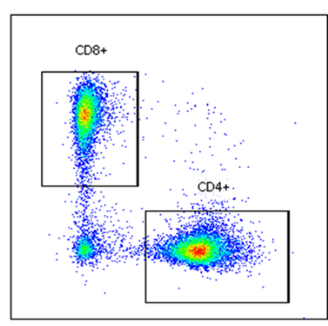


**CD127**

**CD25**


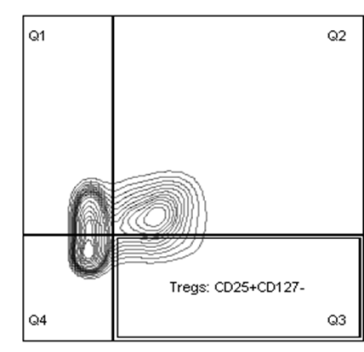


CD25+CD127low/-

B High-fat diet

A Standard diet


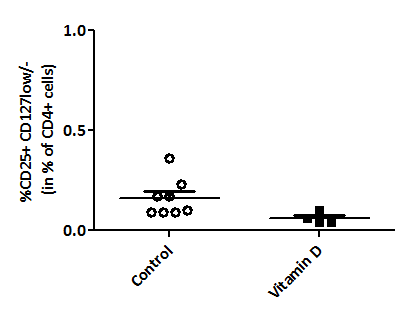


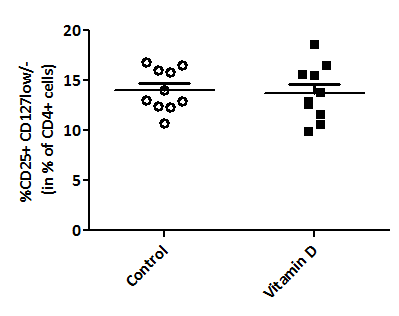


**Vitamin D** **does not affect the presence of CD4+CD25+CD127low/-T cells in the tumor both in basal and high-fat diet conditions.** Flow cytometry analysis of the tumor from Control and Vitamin D-treated mice in basal (A) and high-fat diet (B) conditions. Quantification of the percentage of CD4+ T cell subsets (CD4+CD25+CD127low/-) in the total CD4+ T cell population.
